# Correlation between prognostic nutritional index and oral mucositis in patients with lung adenocarcinoma treated with almonertinib: a multicenter prospective study

**DOI:** 10.3389/fnut.2026.1712518

**Published:** 2026-06-05

**Authors:** Simeng Gao, Jingru Han, Zhenlong Tian, Qian Li, Linhao Xie, Min Su, Qiang Chen, Xinhai Zhu, Jianfu Zhao

**Affiliations:** 1 Department of Oncology, Cancer Diagnosis and Therapy Research Center, The First Affiliated Hospital of Jinan University, Guangzhou, China; 2 Department of Oncology, Shantou Central Hospital, Shantou, China; 3 Department of Oncology, Guangzhou Development District Hospital, Guangzhou, China

**Keywords:** adverse reactions, almonertinib, lung adenocarcinoma, oral mucositis, prognostic nutritional index

## Abstract

Oral mucositis (OM) is a common adverse reaction to epidermal growth factor receptor tyrosine kinase inhibitors (EGFR-TKIs), and severe cases can impair patient compliance and quality of life. The prognostic nutritional index (PNI) comprehensively reflects an individual’s nutritional and immune status and has been applied in lung cancer prognosis studies. However, its relationship with treatment-related OM associated with EGFR-TKI remains unclear. This study aims to investigate the correlation between PNI and the occurrence of OM in patients with lung adenocarcinoma treated with almonertinib.

**Methods:**

This study is a multicenter, prospective observational study. From May 2025 to September 2025, 14,039 patients were assessed for eligibility on the unified platform; 537 met the eligibility criteria and had complete baseline data for analysis. Baseline clinical and laboratory data were collected, and PNI was calculated as 5 × lymphocyte count (10^9^/L) + serum albumin (g/L). OM occurrence was assessed at three-week intervals and graded according to the WHO scoring systems. Independent associations between PNI and OM were evaluated using logistic regression, propensity score matching (PSM), restricted cubic splines (RCS), and subgroup analyses.

**Results:**

During follow-up, 14.5% of patients developed OM. Multivariate logistic regression analysis showed that for each 1-unit increase in PNI, the risk of OM decreased by approximately 1.4% (OR = 0.986, 95% CI: 0.972–1.000). Trend analysis results indicate that patients with high PNI levels exhibit a significantly reduced risk of developing OM (*P* for trend < 0.01). The RCS model suggested a linear negative correlation between PNI and OM. Subgroup analysis results were robust, though interactions were observed across different clinical stages.

**Conclusion:**

Low PNI levels are independently associated with a significantly increased risk of OM. As a simple, cost-effective, and readily accessible indicator, PNI holds promise as a valuable tool for early risk stratification and personalized management in patients with EGFR-mutated lung adenocarcinoma, providing guidance for optimizing targeted therapy throughout the treatment journey.

## Introduction

Lung cancer ranks among the most prevalent and deadly malignant tumors globally and in China. In 2022, approximately 2.48 million new cases were diagnosed worldwide, accounting for 12.4% of all new cancer diagnoses, with about 1.82 million deaths—representing 18.7% of all cancer-related fatalities (1). In China, lung cancer has long ranked first in both incidence and mortality rates among malignant tumors, with approximately 820,000 new cases and 710,000 deaths annually (2). Non-small cell lung cancer (NSCLC) accounts for approximately 80–85% of all lung cancers, with adenocarcinoma being the most common type (3). With advances in molecular biology and genetic testing technologies, it has become increasingly recognized that NSCLC is not a single disease entity but rather a heterogeneous group of tumors characterized by multiple driver gene mutations. Among these, epidermal growth factor receptor (EGFR) gene mutations are one of the most common driver events (4). Common sensitive mutations include exon 19 deletion (19del) and exon 21 L858R point mutation, accounting for approximately 85–90% of EGFR mutations (5). Abnormal activation of EGFR promotes tumor cell proliferation, suppresses apoptosis, and enhances angiogenesis and metastatic potential. Targeted therapy against EGFR mutations, particularly the emergence of third-generation tyrosine kinase inhibitors (TKIs), has become a core treatment strategy for advanced EGFR-mutated NSCLC (6). Among these, almonertinib—China’s independently developed third-generation EGFR-TKI—exhibits high selectivity and irreversible binding characteristics. It not only effectively inhibits sensitive mutations and certain drug-resistant mutations but also reduces common adverse reactions such as rash and diarrhea due to its weaker inhibitory effect on wild-type EGFR. Its favorable oral bioavailability and extended half-life enable once-daily dosing, thereby improving patient compliance. Multiple clinical studies have demonstrated that almonertinib exhibits significant advantages in progression-free survival (PFS) and objective response rate (ORR) among patients with advanced EGFR-mutated NSCLC. In the APOLLO study, among patients with T790M mutation who had previously received first- or second-generation EGFR-TKI therapy, almonertinib demonstrated an ORR of 68.9%, a median PFS of 12.3 months, and a favorable safety profile (7–9).

Although EGFR-TKIs have played a significant role in improving the prognosis of patients with EGFR-mutant NSCLC, treatment-related toxicities remain a key factor limiting therapeutic continuity and adherence. Common adverse events include rash, diarrhea, and abnormal liver function, and some patients require dose reduction or temporary interruption due to intolerable toxicities, potentially compromising disease control. Oral mucositis (OM), a clinically significant adverse reaction associated with EGFR-TKI therapy, has been reported at rates ranging from 13 to 72.1% (10). Clinical manifestations typically include oral mucosal erythema, erosion, or aphthous-like ulcerative lesions. Although high-grade OM is relatively uncommon, it can cause substantial pain and eating difficulties, leading to malnutrition, weight loss, reduced quality of life, and an increased risk of infection (10). In severe cases, OM may necessitate hospitalization or treatment interruption/discontinuation, resulting in suboptimal disease control. However, epidemiological data and risk factors for EGFR-TKI–related OM, particularly among patients treated with almonertinib, remain limited, with a notable lack of systematic evidence in Chinese populations. Therefore, identifying high-risk patients and implementing timely preventive interventions represent critical clinical challenges in optimizing the comprehensive management of EGFR-TKI therapy (11).

The prognostic nutritional index (PNI) has been increasingly used in oncology research because it is simple to calculate, low cost, and reflects both nutritional and immune status. In lung cancer, multiple studies have shown that a lower baseline PNI is strongly associated with poor prognosis. For example, a meta-analysis including 22 studies and 2,550 lung cancer patients treated with immune checkpoint inhibitors demonstrated that low PNI was significantly associated with worse PFS and OS, particularly in the NSCLC subgroup (12). Similarly, in advanced lung cancer receiving first-line immunotherapy, patients with higher PNI had significantly better PFS and OS than those with lower PNI (13). In advanced NSCLC treated with chemo-immunotherapy, PNI has also been reported as an independent prognostic factor for OS and PFS, with a more pronounced difference among patients with low PD-L1 expression (<50%) (14). Moreover, a study of NSCLC patients treated with PD-1 inhibitors found that PNI < 50 was significantly associated with poorer treatment response and lower three-year OS (15). Zhang et al. (16) reported that among patients with extensive-stage small cell lung cancer receiving chemotherapy combined with immunotherapy, those with higher baseline PNI not only had better prognosis but also exhibited higher incidence of immune-related adverse events (IRAEs). Although these findings provide preliminary evidence for the application of PNI in prognosis and adverse reaction prediction, current research on oral mucositis in patients with lung adenocarcinoma treated with EGFR-TKIs remains limited, particularly lacking large-scale, multicenter systematic analyses. Therefore, it is necessary to further explore the predictive value of PNI in this population to achieve early risk stratification, thereby providing evidence-based support for individualized interventions and optimized treatment management.

The objective of this study is to investigate the relationship between PNI and oral mucositis in patients with lung adenocarcinoma treated with almonertinib. By addressing this research gap, this study aims to provide new evidence for the investigation of EGFR-TKI-related adverse reactions and propose a simple tool for early identification and risk stratification in high-risk patients. Ultimately, it seeks to offer guidance for individualized management and optimized treatment strategies.

## Methods

### Data resources

This study is a multicenter, prospective observational cohort study. From May 2025 to September 2025, 14,039 patients with lung adenocarcinoma receiving almonertinib were prospectively assessed for eligibility across multiple medical centers in China, and 537 patients were ultimately selected for analysis in this study. No additional interventions were performed on the patients, and study data were extracted from patient-submitted medical records and related clinical documents uploaded via the unified online platform. The study design and reporting adhere to the “Guidance Principles for the Design and Protocol Framework of Real-World Drug Studies” issued by the Center for Drug Evaluation of the China National Medical Products Administration and the Declaration of Helsinki. It strictly complies with the Personal Information Protection Law and relevant data privacy protection requirements. This study was approved by the Ethics Committee of the First Affiliated Hospital of Jinan University (Approval No.: KY-2024-149), and all patients provided electronic informed consent via the unified online platform. The manuscript preparation adheres to the STROBE statement guidelines.

### Study population and study design

**Figure 1:**
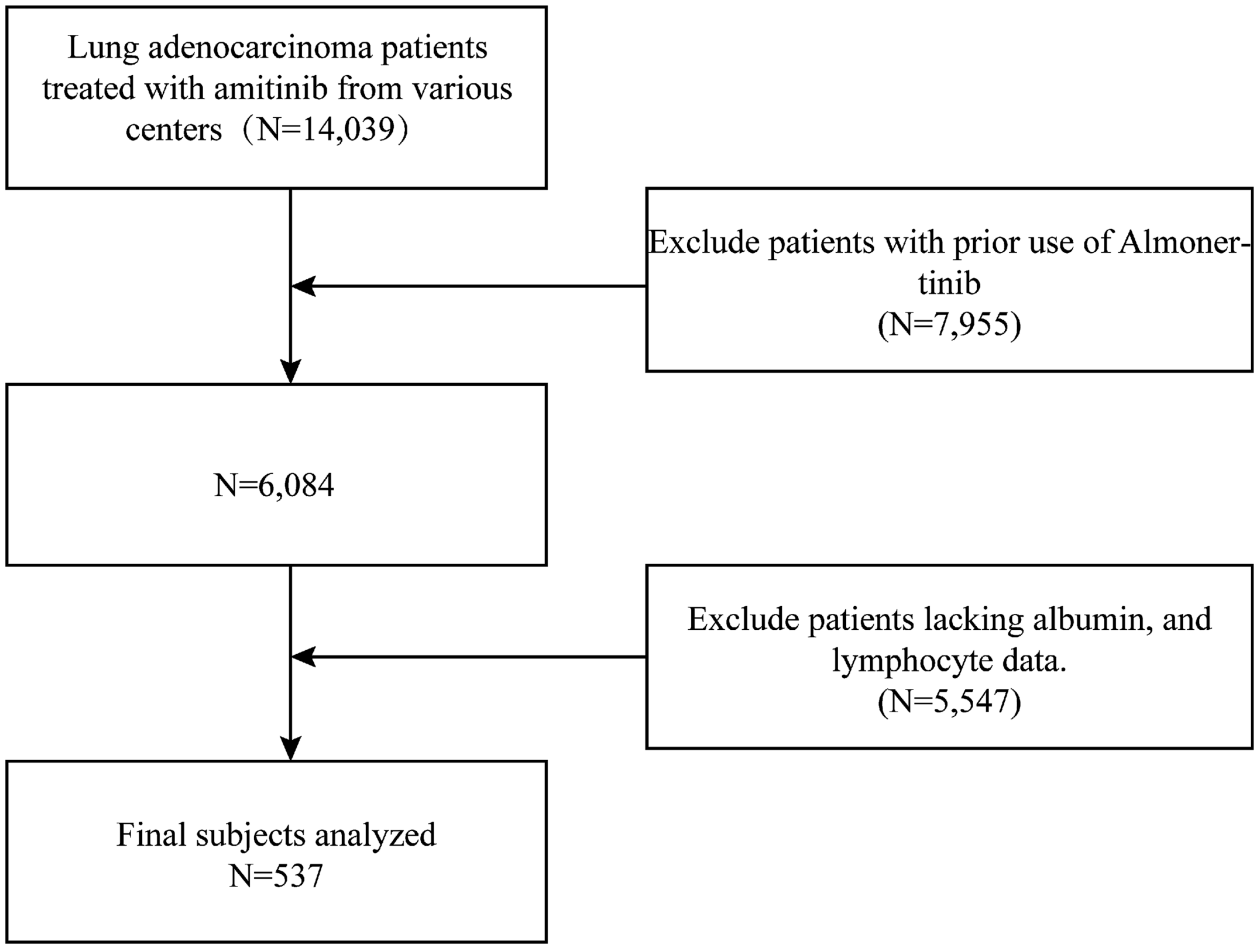
Participant inclusion and exclusion process.

Based on the inclusion and exclusion criteria established for this study, clinical data were systematically collected from patients. All researchers involved in follow-up received standardized training and were responsible for patient screening and data collection. Data collection utilized standardized Excel spreadsheets to enhance data entry efficiency and integrity. Inclusion criteria include: (1) Pathologically confirmed lung adenocarcinoma; (2) Age ≥18 years; (3) First-time treatment with almonertinib with an estimated survival exceeding 3 months; (4) No significant oral mucosal lesions at enrollment. Exclusion criteria are: (1) History of other malignant tumors or concurrent anticancer therapy; (2) Patients receiving immunosuppressive therapy (e.g., post-kidney transplantation or long-term immunosuppressive agents); (3) Patients with severe cardiovascular disease, hepatic or renal insufficiency, or other major systemic diseases; (4) Oral mucosal lesions caused by non-targeted therapy or immunotherapy; (5) Individuals with more than 20% missing clinical data. The participant screening process is illustrated in Figure 1.

### Data management

This study implemented systematic management of missing data to ensure data quality and minimize risk of bias. Cases with missing values for critical variables—such as albumin and lymphocyte count—were excluded due to their strong association with primary outcomes, thereby preserving analytical integrity. Individuals with partial missingness exceeding 20% were also excluded. For the remaining variables with lower missing rates—those below 5% of the total sample and not normally distributed—median imputation was applied.

### Exposure variable assessment

The PNI is a comprehensive indicator that assesses a patient’s inflammatory response, immune function, and nutritional status. It has been widely used to evaluate prognostic risk in various malignant tumors. In this study, PNI serves as the primary exposure variable to reflect the baseline nutritional and immune status of lung adenocarcinoma patients during treatment with almonertinib. The PNI calculation formula is: PNI = 5 × peripheral blood lymphocyte count (10^9^/L) + serum albumin level (g/L).

### Evaluation of outcome variables

This study defines oral mucositis as the outcome variable, characterized by typical symptoms such as redness, swelling, ulcers, or pain in the oral mucosa occurring in patients with lung adenocarcinoma receiving almonertinib treatment. Based on clinical presentation and with assistance from the treatment team, mucosal damage was graded using either the WHO oral mucositis scoring system or the RTOG/EORTC grading system, specifically including mild (Grade 1), moderate (Grade 2), severe (Grade 3), and very severe (Grade 4). The outcome event in this study is defined as the occurrence of ≥ Grade 1 oral mucositis during treatment. Follow-up assessments are conducted every 3 weeks throughout the study period to ensure accurate documentation of symptom onset and progression.

### Definition of covariates

Reviewing previous research findings, we collected factors associated with outcome events and incorporated them into our covariates. Data were collected through standardized questionnaires, techniques, and equipment, including: demographic information (age, gender, race); physical examination measurements (weight, height, BMI, systolic blood pressure, diastolic blood pressure); and laboratory tests (complete blood count and relevant biochemical indicators). Additionally, tumor characteristics encompassed histological type, simplified clinical staging, maximum tumor diameter measurements, and assessments of proximal and distal metastases. To ensure data quality, covariates with high missing values were excluded, retaining only relatively complete variables for subsequent analysis.

### Statistical analysis

All statistical analyses were performed using R software (version 4.3.1). Categorical variables are expressed as percentages, with intergroup differences assessed using weighted chi-square tests. Continuous variables are presented as mean (standard deviation), with differences evaluated using weighted t-tests. To preliminarily explore the association between PNI scores and oral mucositis related to targeted therapy, PNI scores were first grouped by quartiles (Q1–Q4), and differences in oral mucositis incidence across these PNI levels were compared. To identify the features most closely associated with the outcome variable, we first applied two machine learning-based variable selection methods, namely LASSO regression and the Boruta algorithm, to perform preliminary screening of candidate variables. Subsequently, the key variables jointly selected by these two methods were incorporated into a random forest model, and Shapley Additive Explanations (SHAP) values were further used to evaluate and rank the relative importance of each variable. The SHAP values were visualized using swarm plots and bar charts, thereby further identifying the features most strongly associated with the outcome variable. To control for potential confounding effects of covariates, the nearest neighbor matching method within Propensity Score Matching (PSM) was employed to pair samples, achieving covariate balance and further validating the effects of PNI across different outcomes. Additionally, three multivariate logistic regression models were constructed in the original unmatched dataset with stepwise covariate adjustment to assess the independent association between PNI and oral mucositis, and trend tests were performed across PNI quartiles. To explore potential nonlinear relationships between PNI and oral mucositis, a Restricted Cubic Spline (RCS) model was further applied for fitting. Finally, stratified subgroup analyses were conducted to assess the robustness of PNI across different population characteristics, and interaction tests were employed to evaluate the statistical significance of differences between subgroups.

## Results

### Baseline characteristics

This study initially enrolled 14,039 patients with lung adenocarcinoma receiving almonertinib treatment. Following inclusion and exclusion criteria, a total of 537 patients ultimately met eligibility requirements and were included in the analysis. The mean age of the study population was 63.96 years (±10.74), with males accounting for 36.9%. Among all participants, 14.5% were diagnosed with OM (Supplementary Table 1). To preliminarily explore the relationship between PNI levels and oral mucositis, PNI values were divided into four quartile intervals: Q1 (10.10–46.90), Q2 (46.91–50.65), Q3 (50.66–55.20), and Q4 (55.21–320.80). Results indicated that higher PNI levels corresponded to a lower risk of oral mucositis, demonstrating a negative correlation trend. Concurrently, we observed that as PNI levels increased, carcinoembryonic antigen (CEA) levels and clinical staging showed a decreasing trend, while total protein, albumin, and hemoglobin levels gradually increased. All these differences were statistically significant (*p* < 0.05) (Table 1).

**Table 1:** Participant baseline characteristics.

Variables	Q1 (*N* = 134)	Q2 (*N* = 134)	Q3 (*N* = 134)	Q4 (*N* = 135)	*p*-value
Age (year)	64.89 ± 11.34	64.89 ± 10.81	63.72 ± 9.60	62.36 ± 11.02	0.166
BMI(kg/m^2^)	22.78 ± 5.17	22.90 ± 3.80	23.59 ± 5.13	23.74 ± 3.89	0.211
Carcinoembryonic antigen (ug/L)	70.90 ± 203.66	52.33 ± 168.18	40.43 ± 137.07	12.88 ± 44.99	0.015
Alanine aminotransferase (U/L)	20.73 ± 17.89	25.61 ± 30.06	25.63 ± 39.16	24.68 ± 25.16	0.461
Aspartate aminotransferase (U/L)	25.31 ± 28.60	26.34 ± 18.94	27.36 ± 26.36	25.36 ± 12.62	0.862
Alkaline phosphatase (U/L)	76.71 ± 40.54	81.02 ± 35.17	229.66 ± 1057.82	75.14 ± 32.84	0.041
Gamma-glutamyltransferase (U/L)	53.56 ± 115.69	50.79 ± 212.14	27.62 ± 19.14	35.97 ± 78.37	0.29
Total Bilirubin (umol/L)	12.23 ± 13.45	13.62 ± 14.40	12.55 ± 8.86	13.01 ± 5.65	0.759
Total Protein (g/L)	63.68 ± 8.33	70.33 ± 3.99	71.43 ± 5.04	73.09 ± 7.49	<0.001
Albumin (g/L)	35.08 ± 5.49	41.77 ± 1.25	43.74 ± 1.26	46.27 ± 3.11	<0.001
Urea (mmol/L)	9.15 ± 33.96	7.28 ± 21.27	7.85 ± 17.50	7.90 ± 28.66	0.947
Urea nitrogen (mmol/L)	5.95 ± 5.12	7.61 ± 21.74	5.99 ± 5.32	8.22 ± 28.73	0.666
Creatinine (umol/L)	78.50 ± 75.05	72.47 ± 41.43	78.55 ± 48.53	123.92 ± 643.83	0.54
Glucose (mmol/L)	5.52 ± 1.35	5.60 ± 0.98	9.74 ± 32.43	8.47 ± 25.62	0.244
Creatine kinase (umol/L)	190.74 ± 562.88	138.45 ± 90.90	258.16 ± 1065.48	289.16 ± 1301.01	0.506
Leucocyte (10^^9^)	6.50 ± 2.84	6.06 ± 2.10	6.24 ± 2.06	6.65 ± 5.47	0.499
Hemoglobin (g/L)	122.94 ± 20.95	128.04 ± 20.60	131.60 ± 15.78	134.79 ± 16.80	<0.001
Platelets (10^9^)	212.20 ± 83.96	202.75 ± 68.88	196.80 ± 63.48	196.74 ± 64.07	0.233
Sex, %					0.871
Female	81 (60.45%)	84 (62.69%)	87 (64.93%)	87 (64.44%)	
Male	53 (39.55%)	50 (37.31%)	47 (35.07%)	48 (35.56%)	
Race, %					0.274
Others	7 (5.22%)	8 (5.97%)	7 (5.22%)	2 (1.48%)	
Han Race	127 (94.78%)	126 (94.03%)	127 (94.78%)	133 (98.52%)	
Transfer, %					0.152
No	99 (73.88%)	106 (79.10%)	105 (78.36%)	115 (85.19%)	
Yes	35 (26.12%)	28 (20.90%)	29 (21.64%)	20 (14.81%)	
Simplified clinical staging, %					0.004
I	34 (25.37%)	44 (32.84%)	34 (25.37%)	35 (25.93%)	
II	19 (14.18%)	18 (13.43%)	25 (18.66%)	30 (22.22%)	
III	14 (10.45%)	16 (11.94%)	30 (22.39%)	30 (22.22%)	
IV	67 (50.00%)	56 (41.79%)	45 (33.58%)	40 (29.63%)	
Oral mucositis, %					0.012
No	105 (78.36%)	112 (83.58%)	123 (91.79%)	119 (88.15%)	
Yes	29 (21.64%)	22 (16.42%)	11 (8.21%)	16 (11.85%)	

### Assessment of the importance of PNI variables

**Figure 2:**
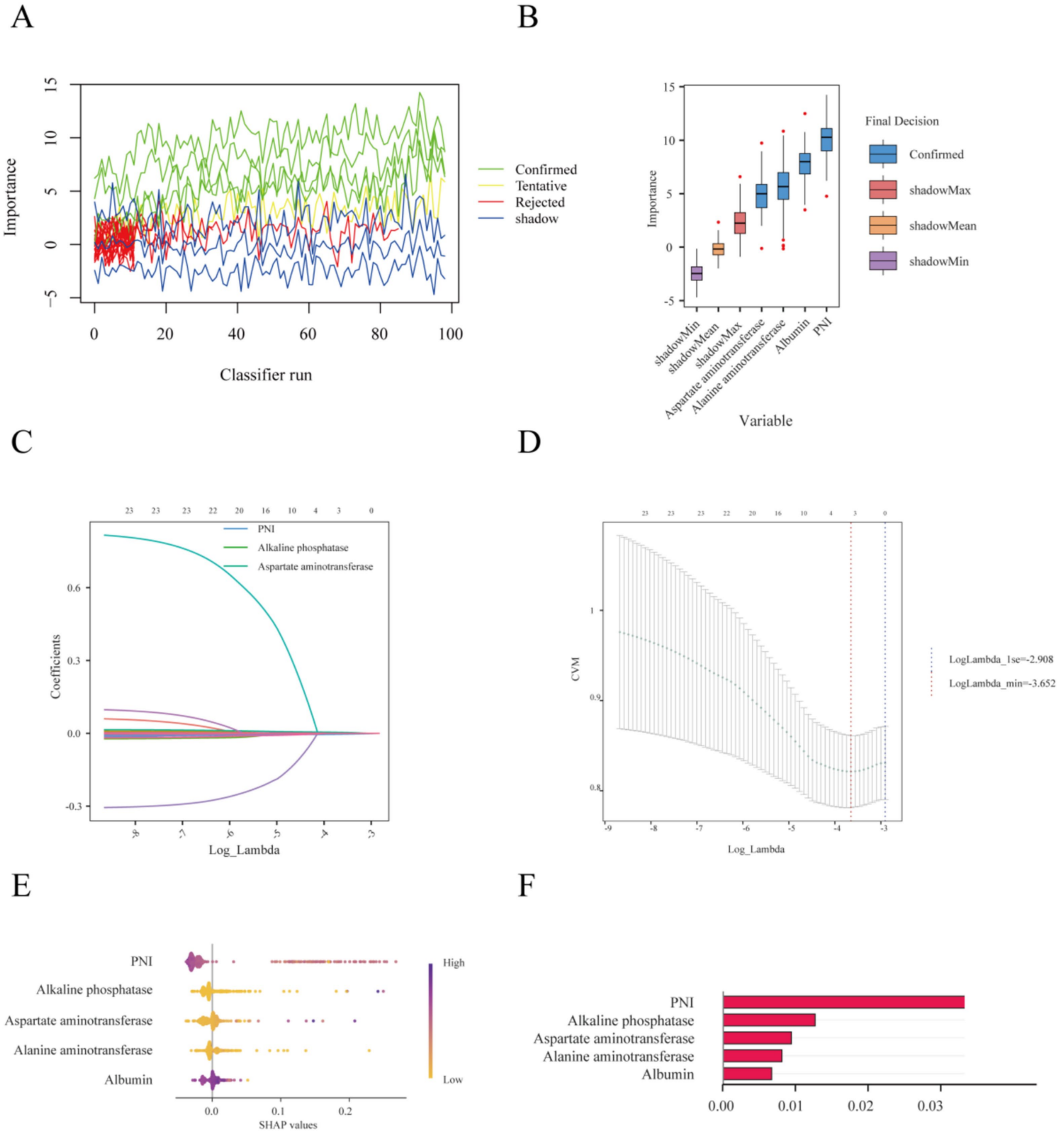
Variable screening results. **(A)** Boruta feature filtering importance score plot. **(B)** Boruta feature filtering each variable importance box plot. **(C)** Lasso regression Lambda and coefficients plot. **(D)** Lasso regression Lambda and CVM plot. **(E)** SHAP bees plot. **(F)** SHAP importance plot.

To further determine the significance of PNI as an exposure variable, this study employed LASSO regression combined with the Boruta algorithm for variable screening. First, the Boruta algorithm was applied to screen all feature variables and assign importance scores (Figure 2A), identifying those variables significantly influencing OM occurrence (Figure 2B): PNI, albumin, alanine aminotransferase, and aspartate aminotransferase. Simultaneously, by integrating LASSO regression and employing 10-fold cross-validation to determine the optimal regularization parameter *λ*, we selected the λ value corresponding to the minimum least-squares bias. This process identified three key variables with non-zero coefficients (Figures 2C,D), including PNI, alkaline phosphatase, and aspartate aminotransferase. Through the aforementioned screening process, five variables were jointly identified by LASSO regression and the Boruta algorithm as potential risk factors associated with OM: PNI, alkaline phosphatase, aspartate aminotransferase, alanine aminotransferase, and albumin. To further evaluate the relative impact of these variables on OM risk, a machine learning-based random forest model was introduced to rank feature importance. Global SHAP explanations in the honeycomb plot (Figure 2E) and bar chart (Figure 2F) revealed that PNI exhibited significantly higher SHAP values than other variables, indicating its greatest weight in the model and establishing it as the primary factor influencing OM occurrence risk.

### Propensity score matching

**Figure 3:**
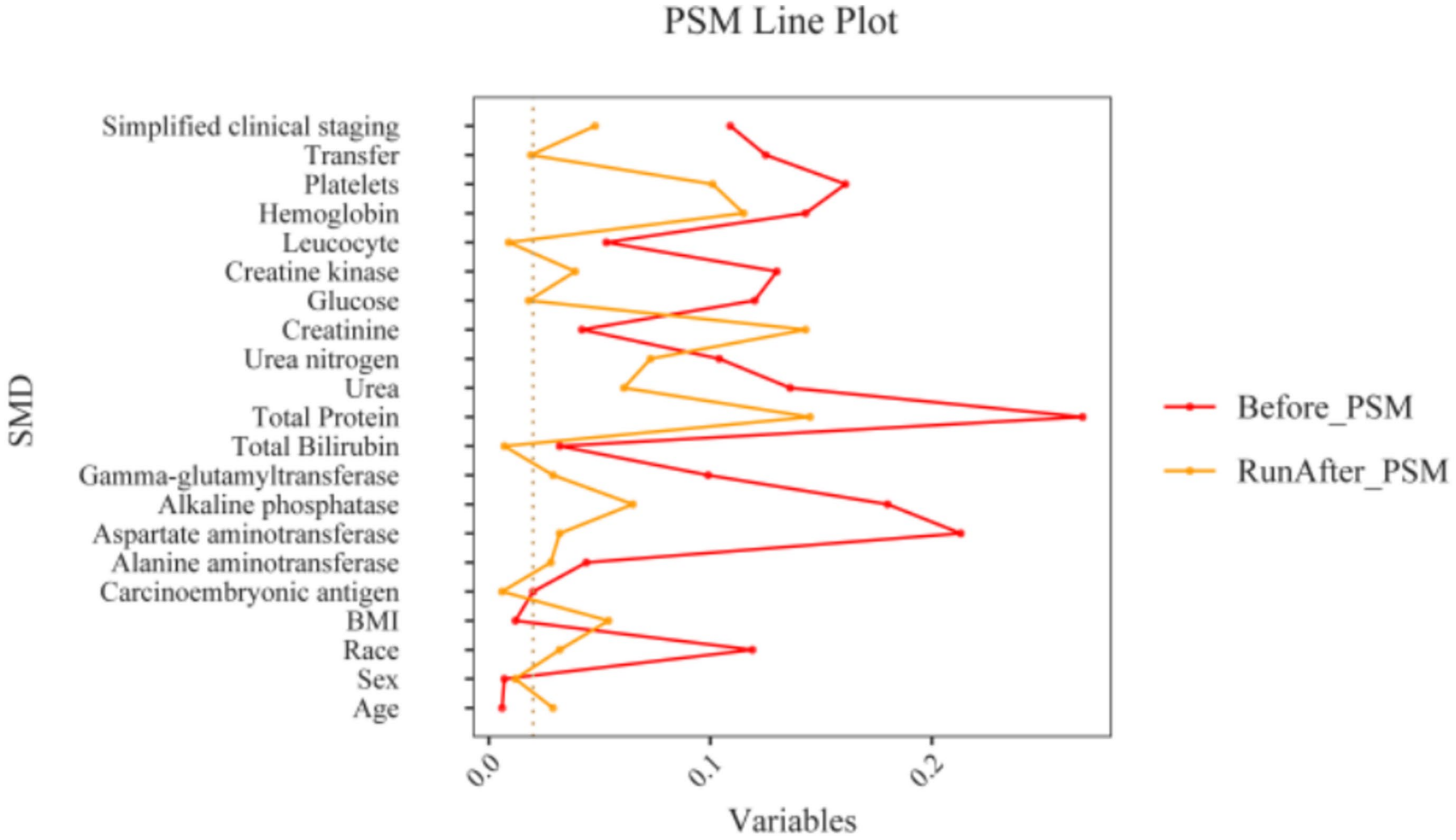
Standardized mean differences of various variables before and after PSM.

To further eliminate the interference of confounding variables on the study results, this research employed PSM using the nearest neighbor method to match the samples. After matching, a total of 75 OM patients successfully matched with control samples. The standardized mean differences (SMDs) following PSM decreased to approximately 0.1, indicating that the matched samples achieved good balance in covariates (Figure 3). Additionally, we compared the matched sets of variable characteristics (Supplementary Table 2). Results showed that, under the premise of basic sample balance, the PNI levels in the OM group were significantly lower than those in the non-OM group, with a statistically significant difference (*p* = 0.007). This finding further supports the strong negative correlation between PNI and the risk of developing oral mucositis.

### Correlation between PNI index and OM

This study constructed a multivariate logistic regression model to evaluate the correlation between the PNI index and the risk of OM. Across three progressively adjusted models, the results consistently demonstrated a statistically significant negative correlation between PNI and OM. Specifically, when PNI was included as a continuous variable in the model, each additional unit of PNI was associated with a 1.4% reduction in the risk of OM (OR = 0.986, 95% CI: 0.972–1.000). Further analysis grouped PNI into quartiles revealed that the risk of OM was significantly lower in the high PNI quartile compared to the lowest PNI quartile. Trend analysis results also support the statistical significance of this negative correlation across different PNI levels (Table 2). In summary, regardless of whether the PNI index is included in the model as a continuous variable or a categorical variable, it consistently exhibits a negative correlation trend with OM risk.

**Table 2:** Correlation between PNI Index and OM.

PNI	Model 1 OR (95%CI)	Model 2 OR (95%CI)	Model 3 OR (95%CI)
Continuous	0.984 (0.969, 1.000)	0.984 (0.969, 1.000)	0.986 (0.972, 1.000)
Categories			
Q1 (<46.90)	1 (reference)	1 (reference)	1 (reference)
Q2 (46.91–50.65)	0.778 (0.417, 1.450)	0.767 (0.410, 1.434)	0.852 (0.429, 1.691)
Q3 (50.66–55.20)	0.599 (0.312, 1.149)	0.588 (0.305, 1.131)	0.636 (0.294, 1.377)
Q4 (≥55.20)	0.348 (0.165, 0.735)	0.337 (0.159, 0.716)	0.352 (0.154, 0.806)
*P* for trend	0.00380	0.00306	0.00962

Model 1 does not adjust for covariates. Model 2 adjusts for sex, age, race, and BMI. Model 3 adjusts for age, sex, race, alanine aminotransferase, aspartate aminotransferase, Alkaline phosphatase, Gamma-glutamyltransferase, Total Bilirubin, Total Protein, Urea, Urea nitrogen, Creatinine, Glucose, Creatine kinase, Leucocyte, Hemoglobin, Platelets, Carcinoembryonic antigen, Transfer, Simplified clinical staging.

### Dose–response association

**Figure 4:**
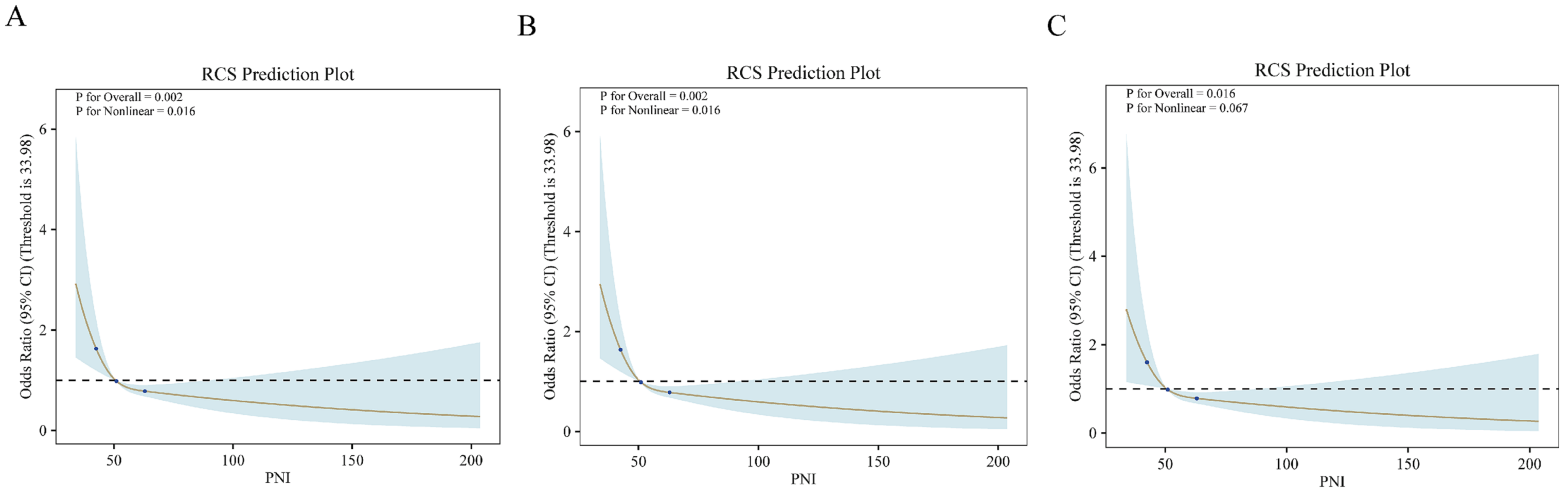
Dose–response relationship between PNI index and OM. **(A)** RCS curve of the unadjusted model; **(B)** RCS curve of the partially adjusted model; **(C)** RCS curve of the fully adjusted model.

To investigate whether a nonlinear dose–response relationship exists between PNI and OM, this study constructed Restricted Cubic Spline models based on the aforementioned three logistic regression models (M1–M3) for analysis. Results showed that in all three models, PNI exhibited a significant negative correlation with OM (*P* for overall < 0.05) (Figure 4). In models with no or partial adjustment for confounding factors (M1 and M2), the relationship between PNI and oral mucositis exhibited a significant nonlinear trend (*P* for Nonlinear < 0.05) (Figures 4A,B). However, after adjusting for all potential confounding factors (Model 3), although a certain nonlinear trend was still observed, this relationship did not reach statistical significance (*P* for Nonlinear > 0.05) (Figure 4C).

### Subgroup analysis

**Figure 5:**
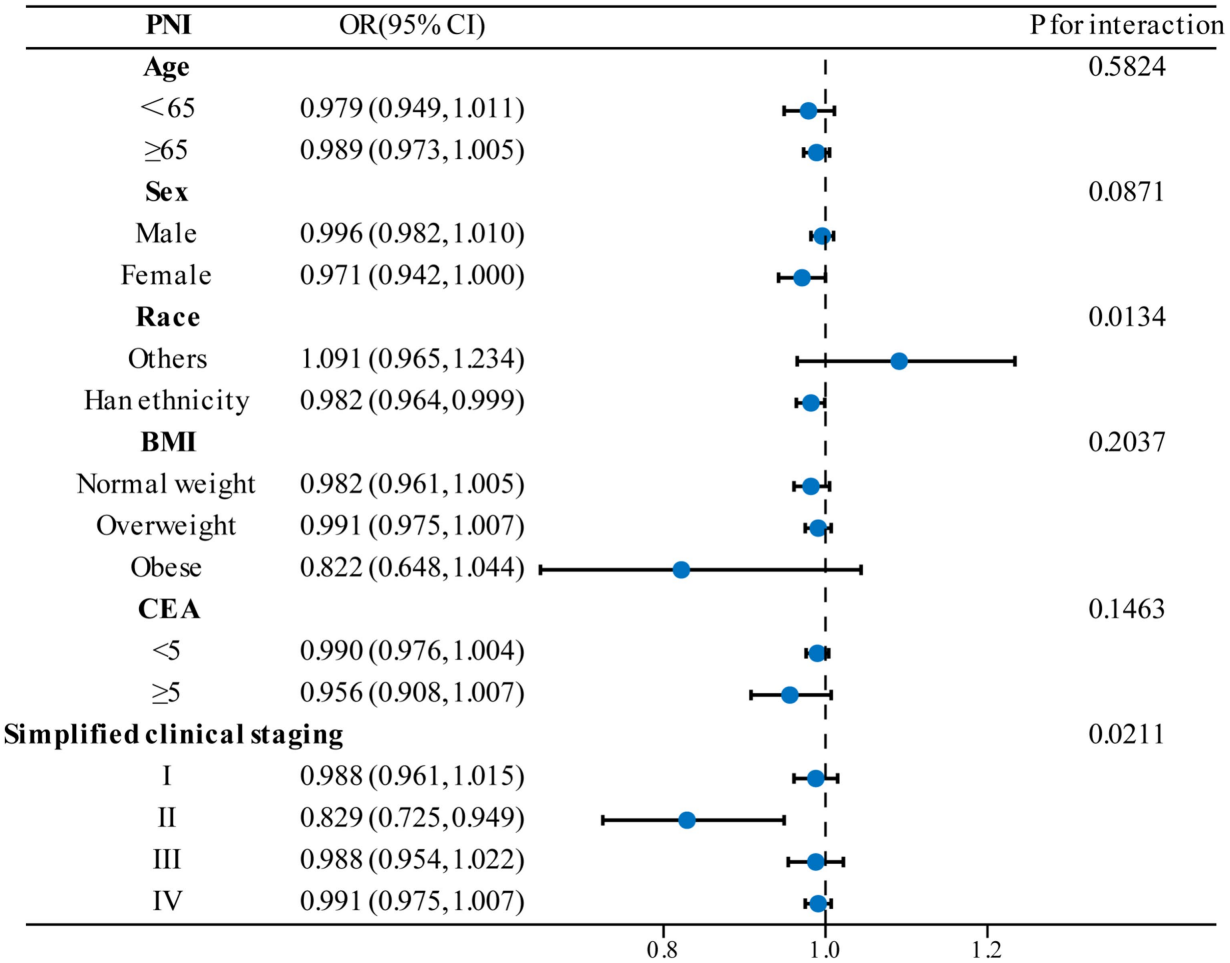
Subgroup analysis of PNI index and OM risk.

To assess the stability and heterogeneity of the relationship between PNI and OM across different demographic characteristics, this study conducted multiple subgroup analyses. Participants were stratified based on variables including age, gender, race, BMI, CEA levels, and tumor clinical stage. Analysis results indicate that the association between PNI and OM continues to show a consistent negative correlation trend across nearly all subgroups, suggesting strong robustness of this relationship across diverse populations. We further conducted interaction tests within each subgroup. Results demonstrate that in most subgroups, the negative correlation between PNI and oral mucositis remains largely unaffected by population characteristics (Figure 5). However, an interaction was observed between clinical stage and racial subgroup (*P* for interaction < 0.05), suggesting that clinical stage and race may modulate the effect of PNI on the risk of oral mucositis.

## Discussion

In this multicenter observational study, a higher baseline PNI was associated with a lower risk of OM among patients with lung adenocarcinoma receiving almonertinib. Results indicate that lower PNI levels correlate with a higher likelihood of oral mucositis in patients. This association remained consistent across multivariable regression, PSM, and subgroup analyses, suggesting that PNI may serve as a useful indicator for assessing the risk of oral mucositis during almonertinib therapy. Furthermore, dose–response analyses suggested an approximately linear inverse association between baseline PNI and the risk of oral mucositis. An apparent non-linear pattern observed in minimally adjusted models was attenuated after stepwise covariate adjustment and was no longer statistically significant. Interaction analyses indicated that the association differed across clinical stages.

Compared with previous studies, the findings of this research exhibit distinct differences and innovative aspects. Most studies have focused on the relationship between PNI and survival outcomes in cancer patients, with a prevailing consensus that lower PNI scores are closely associated with poorer overall survival and progression-free survival, particularly among patients receiving immunotherapy or chemotherapy (12, 15, 17). However, reports on the relationship between PNI and adverse reactions associated with targeted therapies remain limited, particularly lacking systematic analyses of oral mucositis induced by EGFR-TKIs. This study marks the first application of PNI in investigating EGFR-TKI-related oral toxicity, revealing its potential value as an early risk prediction tool and expanding the clinical utility of PNI. These findings not only enhance the significance of PNI in oncology clinical management but also provide novel evidence for the early identification and intervention of adverse reactions to targeted therapies.

Notably, our subgroup analysis revealed that the relationship between PNI and OM exhibits certain heterogeneity across patients with different clinical stages. In early-stage patients, whose immune function and nutritional status remain relatively intact, fluctuations in PNI levels may offer limited explanatory power regarding adverse outcome risk. Conversely, in mid-to-late stage patients, increased tumor burden, heightened catabolism, and sustained inflammatory activation enable PNI to more sensitively reflect dual host imbalances in nutrition and immunity, thereby demonstrating stronger predictive value. This phenomenon has been rarely addressed in previous studies, with most current literature treating PNI as a holistic prognostic factor without stratifying it by disease stage. Therefore, our findings suggest that using PNI as a uniform risk indicator may be limited, and its explanatory power should be evaluated within the context of disease staging.

PNI, composed of serum albumin and peripheral blood lymphocyte count, serves as a simple and sensitive combined nutritional-immunological indicator. It is widely used for screening nutritional risk in cancer patients and assessing disease prognosis (18). Albumin not only reflects the body’s protein synthesis capacity and nutritional reserves, but also participates in key processes such as maintaining colloidal osmotic pressure, regulating oxidative stress, and transporting drug molecules (19). Lymphocytes are the core components of systemic immune function, representing the host’s capacity to respond to infection and injury. Recent studies have shown that nutritional and immune status not only influence treatment efficacy during cancer therapy but are also significantly associated with the risk of developing various treatment-related adverse reactions (20–22). Therefore, a decrease in PNI often indicates the coexistence of malnutrition, immunosuppression, and inflammatory activation.

In this study, patients with lower PNI levels were more prone to developing almonertinib-related OM. From a biological perspective, this association may be jointly related to nutritional deficiency, impaired immune defense, and amplified mucosal inflammation. PNI is calculated from serum albumin and peripheral lymphocyte count; therefore, reduced PNI usually indicates both insufficient protein reserve and impaired cellular immunity. Low albumin levels may reflect malnutrition, decreased protein synthesis, weakened antioxidant capacity, and delayed tissue repair, thereby compromising the integrity and regenerative capacity of the oral mucosal barrier (23, 24). Oral mucositis is a complex biological process involving epithelial injury, oxidative stress, inflammatory cascade amplification, ulceration, and subsequent mucosal healing. In this process, insufficient nutritional reserve may reduce the ability to repair drug-related epithelial damage, while increased oxidative stress may promote the activation of inflammatory pathways such as NF-κB and MAPK/JNK, further inducing the upregulation of pro-inflammatory mediators, including TNF-*α*, IL-1β, IL-6, and COX-2 (25, 26). Meanwhile, peripheral lymphopenia may indicate impaired immune surveillance and weakened local immune regulation, making it difficult for the host to control inflammation and secondary infection after epithelial injury, thereby further aggravating mucosal damage (27). In addition, tumor progression is often accompanied by chronic inflammatory activation and nutritional depletion, which may further reduce PNI levels and form a vicious cycle, making patients more susceptible to oral toxicity (28). The moderating effect of clinical stage observed in our subgroup analysis also supports this hypothesis, suggesting that the predictive value of PNI for OM risk may be more pronounced in patients with greater tumor burden and more evident systemic inflammatory activation.

Our findings are broadly consistent with previous studies on the clinical value of PNI in NSCLC and other malignancies. Existing studies have shown that, among patients with NSCLC receiving immune checkpoint inhibitors, chemotherapy, or chemoimmunotherapy, low PNI is commonly associated with poorer progression-free survival, overall survival, and treatment response (12, 13, 15). However, previous NSCLC studies have mainly focused on prognostic assessment or systemic treatment efficacy, whereas treatment-related oral toxicity has received less attention. Studies in other malignancies also support the potential value of PNI as an indicator of treatment tolerance and adverse-event risk. For example, in patients with head and neck cancer, lower PNI has been associated with increased treatment-related toxicity and a higher risk of severe radiotherapy-induced oral mucositis (29, 30). Therefore, based on previous evidence, our study further suggests that PNI may not only serve as a prognostic indicator in cancer patients but also help identify patients at high risk of EGFR-TKI-related oral mucositis. Taken together, decreased PNI reflects a pathological state characterized by nutritional deficiency, impaired immune function, enhanced oxidative stress, and inflammatory activation, all of which may contribute to the occurrence and exacerbation of almonertinib-related OM.

Although this study targeted oral mucositis as an endpoint for observing treatment-related adverse reactions, its deeper significance lies in revealing the clinical management value of PNI as a nutrition-immunity composite indicator throughout the entire course of lung cancer treatment.

In recent years, PNI has increasingly been recognized not only as a dynamic indicator capable of predicting risk but also as a tool for guiding interventions. Multiple studies indicate that early implementation of individualized nutritional support—including high-protein diets, anti-inflammatory dietary combinations, or oral nutritional supplements—effectively elevates PNI levels, improves immune and nutritional status, thereby reducing the risk of treatment-related adverse events and enhancing patient compliance and quality of life (31, 32). The findings of this study also validate the predictive value of PNI for the occurrence of oral mucositis, suggesting its potential as a key reference for nutritional intervention. Among numerous available clinical blood markers, compared to parameters like NLR (33) that reflect a single inflammatory state or complex assessment systems such as the CONUT (34) score that rely on multiple laboratory tests, PNI demonstrates greater clinical utility and broader applicability due to its straightforward calculation method, accessibility through routine laboratory data, and lower economic cost. Applying PNI to risk screening prior to targeted therapy and to dynamic monitoring during treatment facilitates the timely identification of high-risk individuals. This provides a basis for developing strategies such as nutritional interventions, follow-up frequency, oral care, and patient education. For patients with low PNI, nutritional fortification and intervention plans may be initiated early to reduce the incidence of adverse reactions, prevent treatment discontinuation due to toxic side effects, and thereby enhance treatment continuity and overall resource utilization efficiency.

Although this study employed a prospective observational design, which offers certain advantages in terms of data collection standardization and completeness and provides new evidence for the application of PNI in predicting almonertinib-related oral mucositis, several limitations should be acknowledged. First, as this was not a randomized controlled trial, causal relationships cannot be fully established, and potential confounding factors cannot be completely eliminated. Some factors that may be associated with the occurrence of oral mucositis, such as dietary habits, oral hygiene status, history of prior oral mucositis, and concomitant medications, were not fully controlled in this study. Although we attempted to reduce bias by using methods such as propensity score matching and multivariable adjustment, a certain degree of selection bias and residual confounding may still remain. Second, the follow-up period in this study was relatively short and primarily focused on the early treatment phase, thus failing to adequately assess the predictive value of PNI for long-term adverse reactions and long-term outcomes. Third, all participants in this study were recruited from Chinese centers; therefore, the applicability of our findings to other ethnic groups, geographic regions, and healthcare settings requires further validation. Differences in genetic background, baseline nutritional status, oral care practices, treatment accessibility, and clinical management patterns may influence both PNI distribution and the risk of almonertinib-related oral mucositis. Fourth, because PNI is calculated using serum albumin and peripheral lymphocyte count, patients with missing values for either component were excluded from the final analysis. Although this complete-case approach helped ensure the accuracy of PNI assessment, it may have introduced selection bias if patients with incomplete laboratory data differed systematically from those included in the analysis, particularly in terms of nutritional status, immune function, disease severity, or treatment adherence. In addition, due to missing data in some variables, we were unable to conduct comprehensive sensitivity analyses for other established nutritional or inflammatory indices. Fifth, this study is primarily an association analysis, and its direct guidance for clinical practice remains limited. Future studies should incorporate more clinical indicators and develop visualized predictive models to better guide clinical decision-making. Finally, as a baseline indicator, PNI reflects only the nutritional and immune status at the time of enrollment and fails to dynamically monitor changes during treatment, potentially underestimating its value in risk assessment. Future studies should validate these findings in larger, multiethnic, and international cohorts, extend the follow-up period, incorporate dynamic PNI monitoring, and apply appropriate missing-data handling methods or sensitivity analyses when feasible.

## Conclusion

This multicenter, large-sample study in patients with lung adenocarcinoma confirms that low PNI levels are significantly associated with the risk of developing oral mucositis related to almonertinib treatment. As an inexpensive, simple, and readily accessible indicator, PNI holds promise as an effective tool for clinical risk assessment and personalized management, providing guidance for optimizing the comprehensive management of EGFR-TKI therapy.

## Data Availability

The data analyzed in this study will be made available upon reasonable request. Requests to access these datasets should be directed to JZ, zhaojianfu@jnu.edu.cn.
