# Supplementary material for: Correlation between prognostic nutritional index and oral mucositis in patients with lung adenocarcinoma treated with almonertinib: a multicenter prospective study
Source: Front Nutr. 2026 Jun 5;13:1712518. doi: 10.3389/fnut.2026.1712518 (PMC13279082; doi:10.3389/fnut.2026.1712518)
Supplement: Supplementary file 1 [file Table_1.docx]

Supplementary Table 1: Baseline Characteristics Between Disease and Non-Disease Groups

| Variable | Overall | Non-OM | OM | *P*-value |
| --- | --- | --- | --- | --- |
|  | N = 537 | N = 459 | N = 78 |  |
| Age (year) | 63.96±10.74 | 63.95±10.70 | 64.01±11.00 | 0.964 |
| BMI(kg/m2) | 23.25±4.55 | 23.25±4.61 | 23.30±4.22 | 0.917 |
| Carcinoembryonic antigen (ug/L) | 44.08±151.41 | 43.62±150.39 | 46.75±158.25 | 0.871 |
| Alanine aminotransferase (U/L) | 24.16±29.09 | 23.98±29.54 | 25.22±26.45 | 0.709 |
| Aspartate aminotransferase (U/L) | 26.09±22.47) | 25.06±15.68 | 32.20±44.82 | 0.167 |
| Alkaline phosphatase (U/L) | 115.56±531.96 | 94.32±355.71 | 240.51±1,094.88 | 0.246 |
| Gamma-glutamyltransferase (U/L) | 41.98±127.39 | 43.46±136.16 | 33.26±51.02 | 0.236 |
| Total Bilirubin (umol/L) | 12.85±11.14 | 12.89±11.84 | 12.60±5.44 | 0.725 |
| Total Protein (g/L) | 69.64±7.37 | 69.95±7.06 | 67.81±8.81 | 0.045 |
| Albumin (g/L) | 41.73±5.28 | 42.02±4.79 | 39.99±7.38 | 0.021 |
| Urea (mmol/L) | 8.04±26.08 | 8.44±28.17 | 5.71±2.66 | 0.043 |
| Urea nitrogen (mmol/L) | 6.95±18.39 | 7.16±19.86 | 5.70±2.47 | 0.131 |
| Creatinine (umol/L) | 88.43±326.29 | 89.99±351.94 | 79.24±65.55 | 0.551 |
| Glucose (mmol/L) | 7.34±20.71 | 7.61±22.39 | 5.71±1.27 | 0.073 |
| Creatine kinase (umol/L) | 219.26±888.24 | 232.21±958.43 | 143.06±146.22 | 0.062 |
| Leucocyte (109) | 6.37±3.42 | 6.34±3.56 | 6.51±2.44 | 0.615 |
| Hemoglobin (g/L) | 129.35±19.13 | 129.75±19.13 | 127.01±19.10 | 0.245 |
| Platelets (109) | 202.11±70.66 | 200.35±68.44 | 212.52±82.25 | 0.22 |
| PNI | 435.13±64.18 | 439.22±60.83 | 411.02±77.34 | **0.003** |
| Sex,% |  |  |  | 0.951 |
| Female | 339.00 (63.13%) | 290.00 (63.18%) | 49.00 (62.82%) |  |
| Male | 198.00 (36.87%) | 169.00 (36.82%) | 29.00 (37.18%) |  |
| Race,% |  |  |  | 0.378 |
| Others | 24.00 (4.47%) | 22.00 (4.79%) | 2.00 (2.56%) |  |
| Han ethnicity | 513.00 (95.53%) | 437.00 (95.21%) | 76.00 (97.44%) |  |
| Transfer,% |  |  |  | 0.325 |
| No | 425.00 (79.14%) | 360.00 (78.43%) | 65.00 (83.33%) |  |
| Yes | 112.00 (20.86%) | 99.00 (21.57%) | 13.00 (16.67%) |  |
| Simplified clinical staging,% |  |  |  | 0.854 |
| I | 147.00 (27.37%) | 123.00 (26.80%) | 24.00 (30.77%) |  |
| II | 92.00 (17.13%) | 79.00 (17.21%) | 13.00 (16.67%) |  |
| III | 90.00 (16.76%) | 79.00 (17.21%) | 11.00 (14.10%) |  |
| IV | 208.00 (38.73%) | 178.00 (38.78%) | 30.00 (38.46%) |  |

Supplementary Table 2: Baseline Characteristics Between Disease and Non-Disease Groups After Propensity Score Matching

| **Variable** | Overall | Non-CIOM | CIOM | *P*-value |
| --- | --- | --- | --- | --- |
|  | N = 396 | N = 321 | N = 75 |  |
| **Age (year)** | 63.51±10.95 | 63.45±10.92 | 63.76±11.12 | 0.825 |
| **BMI(kg/m2)** | 23.16±4.33 | 23.12±4.36 | 23.35±4.23 | 0.674 |
| **Alanine aminotransferase (U/L)** | 49.27±164.51 | 49.45±165.52 | 48.49±161.18 | 0.963 |
| **Aspartate aminotransferase (U/L)** | 23.80±28.96 | 23.94±30.23 | 23.19±22.89 | 0.811 |
| **Alkaline phosphatase (U/L)** | 25.16±13.93 | 25.07±13.71 | 25.53±14.95 | 0.808 |
| **Gamma-glutamyltransferase (U/L)** | 107.43±452.16 | 101.35±424.50 | 133.47±557.85 | 0.641 |
| **Total Bilirubin (umol/L)** | 29.22±24.26 | 29.36±24.13 | 28.64±24.93 | 0.823 |
| **Total Protein (g/L)** | 12.33±6.84 | 12.34±7.19 | 12.29±5.14 | 0.949 |
| **Albumin (g/L)** | 69.28±6.59 | 69.47±6.31 | 68.45±7.66 | 0.286 |
| **Urea (mmol/L)** | 5.38±1.78 | 5.36±1.84 | 5.46±1.51 | 0.614 |
| **Urea nitrogen (mmol/L)** | 5.55±3.02 | 5.59±3.31 | 5.41±1.07 | 0.419 |
| **Creatinine (umol/L)** | 72.37±40.35 | 70.96±31.29 | 78.41±66.43 | 0.347 |
| **Glucose (mmol/L)** | 5.67±1.49 | 5.68±1.56 | 5.65±1.15 | 0.877 |
| **Creatine kinase (umol/L)** | 126.63±54.48 | 126.19±50.74 | 128.51±68.61 | 0.783 |
| **Leucocyte (10**9) | 6.43±3.76 | 6.43±4.03 | 6.40±2.26 | 0.929 |
| **Hemoglobin (g/L)** | 128.53±19.28 | 128.95±19.27 | 126.73±19.39 | 0.374 |
| **Platelets (10**9) | 206.50±68.64 | 205.08±65.59 | 212.54±80.59 | 0.458 |
| **PNI** | 58.92±35.33 | 60.71±37.28 | 51.26±24.14 | **0.007** |
| **Sex,%** |  |  |  | 0.926 |
| Female | 250.00 (63.13%) | 203.00 (63.24%) | 47.00 (62.67%) |  |
| Male | 146.00 (36.87%) | 118.00 (36.76%) | 28.00 (37.33%) |  |
| **Race,%** |  |  |  | 0.799 |
| Others | 9.00 (2.27%) | 7.00 (2.18%) | 2.00 (2.67%) |  |
| Han ethnicity | 387.00 (97.73%) | 314.00 (97.82%) | 73.00 (97.33%) |  |
| **Transfer,%** |  |  |  | 0.881 |
| No | 325.00 (82.07%) | 263.00 (81.93%) | 62.00 (82.67%) |  |
| Yes | 71.00 (17.93%) | 58.00 (18.07%) | 13.00 (17.33%) |  |
| **Simplified clinical staging,%** |  |  |  | 0.986 |
| I | 117.00 (29.55%) | 94.00 (29.28%) | 23.00 (30.67%) |  |
| II | 72.00 (18.18%) | 59.00 (18.38%) | 13.00 (17.33%) |  |
| III | 55.00 (13.89%) | 44.00 (13.71%) | 11.00 (14.67%) |  |
| IV | 152.00 (38.38%) | 124.00 (38.63%) | 28.00 (37.33%) |  |
